# Ascorbic Acid Facilitates Neural Regeneration After Sciatic Nerve Crush Injury

**DOI:** 10.3389/fncel.2019.00108

**Published:** 2019-03-21

**Authors:** Lixia Li, Yuanyuan Li, Zhihao Fan, Xianghai Wang, Zhenlin Li, Jinkun Wen, Junyao Deng, Dandan Tan, Mengjie Pan, Xiaofang Hu, Haowen Zhang, Muhua Lai, Jiasong Guo

**Affiliations:** ^1^Guangdong Provincial Key Laboratory of Construction and Detection in Tissue Engineering, Southern Medical University, Guangzhou, China; ^2^Department of Histology and Embryology, Southern Medical University, Guangzhou, China; ^3^Guangzhou Regenerative Medicine and Health Guangdong Laboratory, Guangzhou, China; ^4^Key Laboratory of Mental Health of the Ministry of Education, Southern Medical University, Guangzhou, China

**Keywords:** ascorbic acid, nerve regeneration, peripheral nerve injury, neuron, Schwann cell, macrophage

## Abstract

Ascorbic acid (AA) is an essential micronutrient that has been safely used in the clinic for many years. The present study indicates that AA has an unexpected function in facilitating nerve regeneration. Using a mouse model of sciatic nerve crush injury, we found that AA can significantly accelerate axonal regrowth in the early stage [3 days post-injury (dpi)], a finding that was revealed by immunostaining and Western blotting for antibodies against GAP-43 and SCG10. On day 28 post-injury, histomorphometric assessments demonstrated that AA treatment increased the density, size, and remyelination of regenerated axons in the injured nerve and alleviated myoatrophy in the gastrocnemius. Moreover, the results from various behavioral tests and electrophysiological assays revealed that nerve injury-derived functional defects in motor and sensory behavior as well as in nerve conduction were significantly attenuated by treatment with AA. The potential mechanisms of AA in nerve regeneration were further explored by investigating the effects of AA on three types of cells involved in this process [neurons, Schwann cells (SCs) and macrophages] through a series of experiments. Overall, the data illustrated that AA treatment in cultured dorsal root ganglionic neurons resulted in increased neurite growth and lower expression of RhoA, which is an important inhibitory factor in neural regeneration. In SCs, proliferation, phagocytosis, and neurotrophin expression were all enhanced by AA. Meanwhile, AA treatment also improved proliferation, migration, phagocytosis, and anti-inflammatory polarization in macrophages. In conclusion, this study demonstrated that treatment with AA can promote the morphological and functional recovery of injured peripheral nerves and that this effect is potentially due to AA’s bioeffects on neurons, SCs and macrophages, three of most important types of cells involved in nerve injury and regeneration.

## Introduction

Although the peripheral nervous system (PNS) has an intrinsic ability to repair and regenerate, this capability is limited, and spontaneous neural regeneration is notably slow. Functional recovery from peripheral nerve injury (PNI) is generally far from satisfactory. Due to the slow rate of axonal regeneration, irreversible damage to the structure and function of target organs may occur before regenerated axons reinnervate these targets, which could have a profound and permanent impact on patients (Grinsell and Keating, 2014; Faroni et al., 2015). To alleviate the suffering of PNI patients, research on approaches to accelerate peripheral nerve regeneration is encouraged. In the present study, the effect of ascorbic acid (AA) on neural regeneration after PNI was assessed.

AA, or vitamin C, is a dietary essential micronutrient that plays key roles in many important biological processes (Granger and Eck, 2018). AA is not only considered an important daily nutrient for maintaining health but also has long been used safely in the clinic to protect against many diseases. Moreover, recent studies indicate that AA has many unexpected newly discovered biological functions. For example, AA can be used to promote stem cell reprogramming (Wang et al., 2011; Cimmino et al., 2018). It is essential for the development and physiological functions of nervous tissue (May, 2012; Granger and Eck, 2018) and has therapeutic effects in neurodegenerative diseases (Kocot et al., 2017; Moretti et al., 2017). Clinical studies and animal experiments have demonstrated that AA administration can improve functional recovery from spinal cord injury (Lamid, 1983; Robert et al., 2012; Yan et al., 2014; Guo et al., 2018). Existing data on PNI indicate that AA has antinociceptive effects on PNI animals, which may be achieved mainly through interactions with N-Methyl-D-aspartate (NMDA) receptors or through ROS activity (Lu et al., 2011; Saffarpour and Nasirinezhad, 2017). However, evidence regarding whether AA has an effect on the morphological and functional recovery of injured nerves is lacking. To address this issue, we developed a sciatic nerve crush injury mouse model of PNI to test the potential role of AA in peripheral nerve regeneration. Its potential mechanism was studied in neurons, Schwann cells (SCs) and macrophages, which are the most important cell types involved in nerve injury and regeneration.

## Materials and Methods

### Ethics Statement

All procedures involving animals, including surgery, electrophysiological tests, behavior tests and tissue collection, were carried out with the approval of the Southern Medical University Animal Care and Use Committee in accordance with the guidelines for the ethical treatments of animals. All efforts were made to minimize the number of animals used and their suffering.

### Sciatic Nerve Injury Model Preparation and Drug Administration

C57BL/6 mice (*n* = 16 per group, female, 6–8 weeks old, weighing 18–20 g, provided by the Animal Center of the Southern Medical University) were used for the sciatic nerve crush injury model as described in our previous report (Qian et al., 2018). Briefly, the mice were anesthetized with an intraperitoneal injection of 12 mg/ml tribromoethanol (180 mg/kg body weight). The sciatic nerve in the left leg was bluntly exposed after performing a lateral skin incision along the length of the femur. Then, wound closure without manipulation of the nerve was performed (sham-surgery group), or the mice were subjected to a calibrated sciatic crush injury 0.5 cm distal to the sciatic notch. The nerves were crushed with a fine, smooth, straight hemeostat (tip width: 1 mm) for 2 min, and the crush site was marked with a 9–0 nylon suture as previously reported (Sheu et al., 2012). The mice that underwent nerve injury were randomly divided into the AA and saline groups. AA (purchased from Baiyunshan Pharmaceuticals Company, Guangzhou, China) was prepared in a suspension with saline at a concentration of 13.33 mg/ml. Immediately after surgery, the animals in the AA group received an intragastric administration (i.a.) of the AA solution (400 mg/kg) followed by daily i.a. of AA (200 mg/kg). The mice in the saline group received the same volume of saline as the volume of AA suspension administered to the AA group. The drug administration method strictly followed published protocols (Yagi et al., 2015; Liu et al., 2016). A solution of AA or saline was intragastrically administered *via* a customized probe through which the solution could be pushed forward into the stomach. All animals received routine postoperative care and were housed under standard laboratory conditions with a 12 h/12 h light-dark cycle and free access to food and water. At 3 days post-injury (dpi), six mice per group were sacrificed, and axonal regeneration in the injured nerve was detected by immunohistochemistry and Western blotting for antibodies against growth-associated protein 43 (GAP43) and superior cervical ganglion 10 (SCG10). At 28 dpi, 10 mice per group were subjected to behavioral tests and an electrophysiological assessment. Then, the sciatic nerves and gastrocnemius muscles were collected, and immunohistochemistry, eletromicroscopy, and hemeatoxylin staining were performed as described in the following sections.

### Behavioral Tests

The motor and sensory functional recovery of the injured hindlimb of mice receiving sciatic nerve crush injury was detected at 28 dpi by gait analysis, rotarod, and the hot plate test, which are widely used in sciatic nerve crush injury models (Jungnickel et al., 2010; Gallaher and Steward, 2018). Each mouse was allowed at least a 2-h break between testing sessions. All behavior tests were formally performed 28 dpi, but the mice were trained for 2 days before the final tests.

#### Gait Analysis

Motor function of the experimental mice was quantified using a gait analysis system (Mouse Specifics Inc., Quincy, MA, USA, DigiGait; Glajch et al., 2012). The mice were trained to walk on a motorized transparent treadmill belt, and their footprints were captured with a video camera set beneath the belt. Then, the sciatic functional index (SFI) was calculated with software following the protocol of the system (Jungnickel et al., 2010; Wang et al., 2018).

#### Rotarod Assay

Motor performance was also measured using a rotarod device (TSE Systems; Singh et al., 2017). Briefly, the mice were pretrained for 2 days on an automated 6-lane rotarod unit that could be set at a fixed or accelerating speed. During the training protocol, the mice were placed on the rod as it rotated at a speed of 5 rotations per minute (RPM) for 60 s. During the testing phase, the mice were placed on the rod as it accelerated from 0 RPM to 40 RPM over a period of 20 s. The length of time that each mouse stayed on the rotating rod was measured and recorded.

#### Hot Plate Test

The recovery of sensory function of the injured nerve was assessed by a hind paw thermal withdrawal test using a hot-plate instrument (Muromachi Kikai, Tokyo, Japan) as previously described (Gallaher and Steward, 2018; Ibrahim et al., 2018). Before performing the test, each mouse was habituated twice to the hot plate set to room temperature. Then, each mouse was placed on the hot plate set to a temperature of 55 ± 0.5°C. The withdrawal latency, manifested by the time (in seconds) until hind paw licking or jumping, was recorded.

### Electrophysiological Assessment

Two hours after the final behavioral analyses, each mouse was anesthetized by an intraperitoneal injection of 12 mg/ml tribromoethanol (180 mg/kg body weight) and subjected to electrophysiological tests following a previously described protocol (Wang et al., 2014). Briefly, the sciatic nerve was re-exposed, a pair of stimulating electrodes (13 mm long, 0.5 mm in diameter) was inserted 3 mm proximal to the crushed site to stimulate the sciatic nerve, and a pair of needle electrodes (13 mm long, 0.5 mm in diameter) was inserted subcutaneously into the middle of the intrinsic foot muscle to record the compound muscle action potential (CMAP) with a set of electrophysiological recorders (Axon Digidata 1550 Digitizer, Molecular Devices). The amplitude and latency of each test were analyzed to determine the nerve conduction strength and nerve conduction speed, respectively.

### Tissue Collection

Neural regeneration after nerve injury was detected at two time points. At 3 dpi, six mice per group were sacrificed by decapitation. Then, tissue was collected for Western blotting (*n* = 3 per group) or, following perfusion, for immunohistochemistry (*n* = 3 per group). At 28 dpi, immediately after the electrophysiological recordings were completed, the mice (*n* = 10 per group) were intracardially perfused with 0.1 M phosphate-buffered saline (PBS) for 10 min, followed by 4% paraformaldehyde (PFA, in 0.1 M PBS) for 30 min; then, the sciatic nerves and gastrocnemius muscles were harvested for further investigation.

### Western Blotting

Three mice per group were decapitated at 3 dpi, and a 1-cm piece of the sciatic nerve was dissected from the injury site, frozen in liquid nitrogen for 30 s, minced and homogenized in RIPA lysis buffer (Sigma, St. Louis, MO, USA) containing 1% protease inhibitor cocktail (Cell Signaling). The cultured SCs and macrophages were also homogenized in RIPA lysis buffer (Sigma, St. Louis, MO, USA) containing 1% protease inhibitor cocktail (Cell Signaling). The proteins were separated on 10% sodium dodecyl sulfate-polyacrylamide gels and transferred to polyvinylidene difluoride membranes (Bio-Rad, Hercules, CA, USA). After blocking with 5% bovine serum albumin (BSA) in Tris-buffered solution containing 0.5% Tween-20 for 2 h, the blots were probed overnight at 4°C with the following primary antibodies: rabbit anti-GAP43 (1:2,000; ab16053, Abcam), rabbit anti-SCG10 (1:1,000; NBP1-49461, Novusbio), rabbit anti-iNOS (1:1,000; ab178945, Abcam), rabbit anti-CD163 (1:1,000; ab213612, Abcam), rabbit anti-NGF (1:1,000; ab6199, Abcam), rabbit anti-NT-3 (1:500; sc-547, Santa Cruz), rabbit anti-GDNF (1:500; ab119473, Abcam), rabbit anti-BDNF (1:500; ab6201, Abcam), rabbit anti-laminin (1:500; ab7463, Abcam), rabbit anti-β-actin (1:1,000; ab008, Multisciences). After incubation with an HRP-conjugated secondary antibody (Molecular Probes) for 2 h at room temperature, the immunoreactive proteins were visualized by an enhanced chemiluminescence reaction, and the band density was calculated using Image-Pro Plus 6.0 software (Media Cybernetics).

### Immunohistochemistry

The nerves dissected from the perfused animals (3 dpi, *n* = 3; 28 dpi, *n* = 6 per group) were post-fixed in 4% PFA for 24 h, cryoprotected in 30% sucrose overnight at 4°C, and then sectioned on a cryostat (Leica) at a thickness of 10 μm. The sciatic nerve samples collected at 3 dpi were longitudinally cut 1.5 cm distal to the injury site. The nerves collected at 28 dpi were transversely cut in the range of 5–6 mm distal to the injury site. All sections were mounted onto poly-lysine-coated glass slides and stored at −20°C for immunohistochemistry. Briefly, the sections were penetrated with 0.5% Triton X-100 (Sigma, St. Louis, MO, USA) for 30 min and incubated with blocking buffer [5% fish gelatine (Sigma, St. Louis, MO, USA) containing 0.3% Triton X-100] at room temperature for 1 h, followed by incubation with primary antibodies diluted in blocking buffer overnight at 4°C, incubation with Alexa Fluor 488 and/or 568 fluorescent-conjugated secondary antibodies (Molecular Probes) for 2 h at room temperature, and incubation with DAPI (1:5,000; Sigma, St. Louis, MO, USA) for 2 min to counterstain the nuclei. After immunohistochemistry, the sections were mounted using anti-fading mounting medium (Vector), and images were captured under a fluorescence microscope (Leica). The following primary antibodies were used for immunostaining of the longitudinal sections: rabbit anti-GAP43 (1:400; ab16053, Abcam), rabbit anti-SCG10 (1:500; NBP1-49461, Novusbio), rat anti-mouse f4/80 (1:400; CL89170AP, Cedarlane), rabbit anti-iNOS (1:1,000; ab178945, Abcam) and rabbit anti-CD163 (1:1,000; ab213612, Abcam). In addition, mouse anti-MBP (1:200; NE1018, Calbiochem) and rabbit anti-NF (1:800; N4142, Sigma, St. Louis, MO, USA) primary antibodies were used for double staining of the transverse sections. The number of S100-positive cells, f4/80-positive cells, iNOS-positive cells or CD163-positive cells in the longitudinal sections of each sciatic nerve was counted in a 250 μm × 250 μm area 1 mm distal to the crush site. The number of NF-positive axons in the cross-sections of each sciatic nerve was counted with Photoshop 5.0 software (Adobe, USA) by an investigator blinded to the experimental groups (Schreiber et al., 2015).

### Transmission Electron Microscopy

Partial sciatic nerves dissected at 28 dpi (*n* = 4 per group) were subjected to transmission electron microscopy (TEM). Briefly, 2-mm long segments from an area 5 mm distal to the injury site were post-fixed with 2.5% glutaraldehyde plus 2% PFA for 24 h followed by 1% OsO_4_ for 2 h at 4°C, dehydrated stepwise in increasing concentrations of acetone and embedded in Spurr’s resin (Sigma, St. Louis, MO, USA) for ultrathin transverse sectioning (70 nm). After staining with 2% uranyl acetate and lead citrate, ultrathin sections were observed under a transmission electron microscope (H-7500, Hitachi). Six images of each sample were randomly captured at a magnification of 3,500. Then, the diameter of each axon or whole nerve fiber and the area of each axon were measured with Image-Pro Plus 6.0 software (Media Cybernetics). Based on the collected data, the G-ratio and axon area distribution were calculated to reflect the myelin thickness and axon size, respectively. Briefly, the G-ratio was determined as the ratio of the axon diameter to the fiber diameter (Wen et al., 2017). The axon area distribution was determined by Penna’s protocol (Penna et al., 2011) with minor revisions. Briefly, after the area of each axon was measured by Image-Pro Plus 6.0 software, the collected data were grouped in 5-μm^2^ increments (i.e., 0–5 μm^2^, 5–10 μm^2^, 10–15 μm^2^, etc.). Then, the percentage of the number of axons in each group compared with the total number of axons was determined as the axon area distribution (%).

### Histomorphometry of the Gastrocnemius Muscle

Since the level of myoatrophy in target muscles is known to be an important index of nerve injury and regeneration, we performed histomorphometry of the gastrocnemius muscle at 28 dpi. Briefly, the midbellies of the gastrocnemius muscles from the perfused mice were trimmed and post-fixed with 4% PFA for 24 h at 4°C. Subsequently, the muscles were embedded with Tissue OCT-Freeze Medium and transversally cut into 10-μm sections. The sections were stained with 0.5% hemeatoxylin to show the outline of the myofibers, and the areas of the myofibers were calculated as described in our previous publications (Wang et al., 2014; Pan et al., 2017). Briefly, six random non-overlapping fields from five sections from each animal were captured, and the image analysis was performed using Image-Pro Plus 6.0 software to quantify the areas of the myofibers.

### Primary Cell Culture

#### DRG Neuron Cultures

Based on the protocol described previously (Chang et al., 2016), the dorsal root ganglia (DRG) from neonatal 1-day-old SD rats were rapidly dissected and digested with 0.125% trypsin (Sigma, St. Louis, MO, USA) at 37°C for 30 min. The separated cells were cultured in Dulbecco’s modified Eagle’s medium/Ham’s F-12 (DMEM/F-12) with 1% fetal bovine serum (FBS; Corning) and plated onto the Poly-L-lysine (PLL, Sigma-Aldrich, St. Louis, MO, USA)-treated coverslips in culture dishes. Medium for half of the cultures was supplemented with AA (200 μM, Sigma, St. Louis, MO, USA). After being cultured for 24 h, the cells were fixed with 4% PFA. Immunocytochemistry for a mouse anti-Tuj1 antibody (1:400; T8660, Sigma, St. Louis, MO, USA) or a mouse anti-RhoA antibody (1:200; sc-418, Santa Cruz) was performed following routine protocols.

#### Primary Schwann Cell Cultures

As described in our previous publication (Wen et al., 2017), SCs were isolated from the spinal nerves of SD rats at postnatal day 3. The collected nerves were dissociated with 0.25% trypsin-EDTA (Gibco) at 37°C for 30 min, and single cells suspended in DMEM/F12 (Corning) containing 10% FBS (Corning) were plated onto PLL-coated Petri dishes. After overnight incubation, the cultures were treated with cytosine arabinoside (10 μM, Sigma-Aldrich, St. Louis, MO, USA) for 48 h to eliminate fibroblasts. Then, the cells were routinely cultured with SC medium [DMEM/F12 containing 3% FBS, 3 μM forskolin (Sigma-Aldrich, St. Louis, MO, USA), 10 ng/ml heregulin (PeproTech) and 100 mg/ml penicillin-streptomycin (Gibco)] to expand the cells.

#### Primary Macrophage Cultures

Resident peritoneal macrophages were isolated from adult SD rats. Immediately after the rat was anesthetized by overdose with 80 mg/kg pentobarbital sodium, 10 ml of sterile Hank’s Balanced Salt Solution (HBSS, Gibco) was gently injected into the caudal half of the peritoneal cavity, and then the HBSS containing the resident peritoneal cells was slowly withdrawn. The cells were resuspended in DMEM culture medium (Gibco) with 10% FBS and plated onto dishes at a density of 2 × 10^6^ cells/ml. The medium was replaced twice every 2 h to remove the non-adherent cells. Thereafter, the adhered macrophages were cultured using a routine cell culture protocol (Yuan et al., 2017).

### Cell Proliferation Assays

The proliferating capability of SCs and macrophages was measured by a WST-1 assay and a bromodeoxyuridine (BrdU) assay.

First, a WST-1 Cell Proliferation Assay Kit II (Abcam) was utilized to detect cell proliferation based on the manufacturer’s instructions and previous literature (Wu et al., 2017). Briefly, primary SCs or macrophages were seeded in 96-well plates at a density of 1 × 10^4^ cells/well in 100 μl of medium (SC medium for SCs and DMEM/F12 with 10% FBS for macrophages) with or without 200 μM AA. Following 36 h of routine incubation, WST working solution was added to the culture medium (10 μl/well) and maintained for another 3 h. Then, the absorbance was recorded at 450 nm with a Microplate Reader (Bio-Rad, Hercules, CA, USA). All experiments were performed in triplicate, and three independent experiments were performed.

For the BrdU assay, 1 × 10^5^ SCs or macrophages in 1 ml of medium (SC medium for SCs and DMEM/F12 with 10% FBS for macrophages) with or without 200 μM AA were plated onto PLL-coated coverslips in 24-well plates. Twenty-four hours later, BrdU (10 μM, Sigma, St. Louis, MO, USA) was added to the medium, and the cells were further incubated for 36 h. Then, the cells were fixed with 4% PFA for 1 h, denatured with 2 N HCl at 37°C for 30 min and neutralized with 0.1 M borate buffer (pH = 8.5) for 15 min. Immunocytochemistry was performed as follows: after blocking with 1% BSA for 1 h, the cells were penetrated with 1% Triton X-100 for 1 h, incubated with a mouse anti-BrdU primary antibody (1:200; NA61, Sigma, St. Louis, MO, USA) followed by an Alexa Fluor 568-conjugated secondary antibody (Molecular Probe) and incubated with DAPI (1:5,000, Sigma, St. Louis, MO, USA) for 5 min to counterstain the nuclei of the cells. Five images of each coverslip (the center and four quadrants) were captured using a fluorescence microscope (Leica), and the percentage of cells that were positive for BrdU was quantified. All experiments were performed in triplicate, and three independent experiments were performed.

### Migration Assay

The migration of the SCs and macrophages was assessed by a Transwell assay using 6.5-mm Transwell chambers (8-μm pores, Corning Costar) as described previously (Wen et al., 2017). After the chambers were pretreated with 10 μg/ml laminin solution or culture medium, 1 × 10^5^ SCs or macrophages in 100 μl of DMEM/F12 containing 1% FBS were seeded into the upper chamber, and the lower chamber was filled with 600 μl DMEM/F12 containing 10% FBS without cells. The SCs and macrophages were allowed to migrate for 4 h and 24 h, respectively, and then fixed with 4% PFA for 20 min. After careful removal of the cells on the upper surface with a cotton swab, the cells adhered to the lower surface of the Transwell membrane were stained with 0.1% crystal violet (Beyotime) for 30 min. Then, five images of each membrane (the center and four quadrants) were captured under an inverted microscope (Leica) for quantification.

### Phagocytic Capability Assay

Phagocytosis in SCs and macrophages was assessed by the ingestion of lumispheres. Briefly, 0.1 mg/ml fluorescent lumispheres (1-μm diameter, BaseLine Chromtech, China) were added into the culture medium of SCs or macrophages for 6 h or 3 h, respectively. To accurately count the number of intracellular lumispheres, the lumisphere-treated cells were collected by trypsinization and then re-cultured on PLL-coated slides for 3 h after being rinsed three times with HBSS to remove the attached lumispheres from the cell surface. Finally, the cells were fixed with 4% PFA and stained with CNPase (1:400; BS3461, Bioword Technology, Inc., St. Louis Park, MN, USA) or ED1 (1:200; MAC341GA, AbD Serotec) to identify the outlines of the SCs and macrophages. With a fluorescence microscope (Leica), the number of lumispheres ingested by each cell was counted.

### Statistical Analysis

Statistical analyses were performed using SPSS statistical software 20.0 (IBM). All values are presented as the mean ± the standard error of the mean (SEM). In experiments with two groups, independent samples *t*-tests were used to analyze values between the two groups. In experiments with more than two groups, one-way analysis of variance (ANOVA) was used. A *p*-value <0.05 was considered statistically significant. For all graphs, *indicates *P* < 0.05 and N.D. indicates no significant difference.

## Results

### AA Accelerates Axonal Regeneration in the Early Stage of Nerve Injury

**Figure 1:**
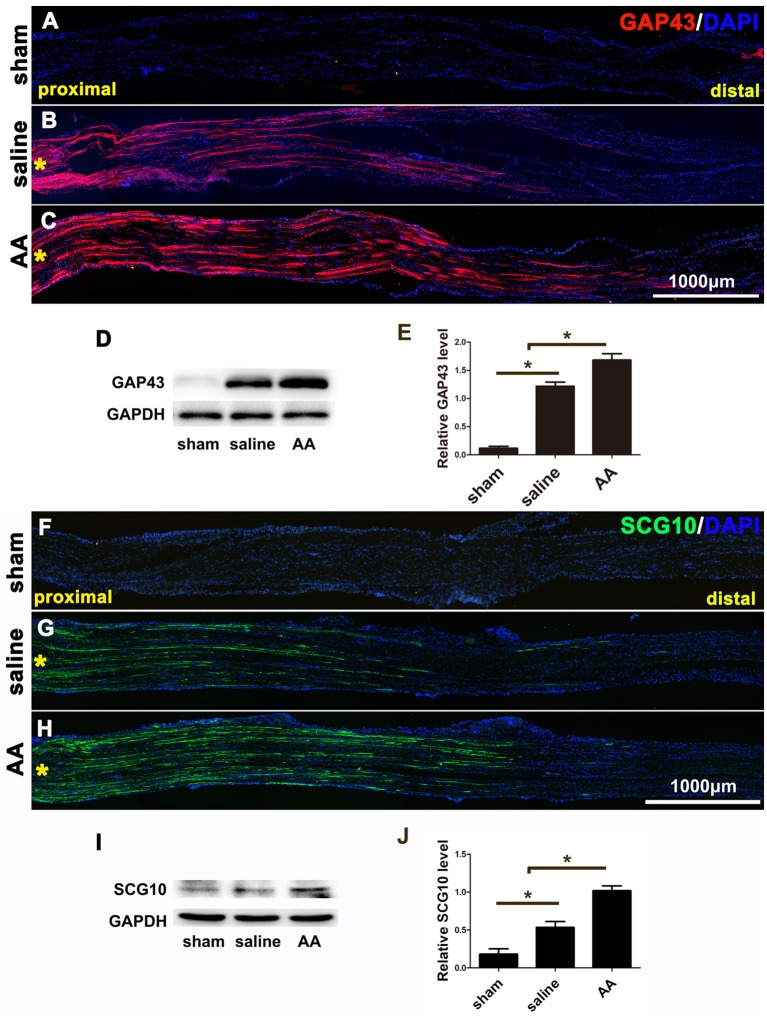
GAP43 and SCG10 immunostaining and Western blotting show that ascorbic acid (AA) accelerates the regrowth of injured axons in the early stage after sciatic nerve crush injury. **(A–C)** Representative samples of GAP43-immunostained longitudinal sciatic nerve sections at 3 dpi from the sham group **(A)**, saline group **(B)**, and AA group **(C)**. Asterisks represent the lesion site. **(D,E)** Western blots and schematic diagrams of the statistical analysis showing the protein level of GAP43 in the distal trunk of the injured sciatic nerves (*n* = 3, *P < 0.05). **(F,G)** Representative samples of SCG10-immunostained longitudinal sciatic nerve sections from the sham group **(F)**, saline group **(G)**, and AA group **(H)**. Asterisks represent the lesion site. **(I,J)** Western blots and schematic diagrams of the statistical analysis showing the protein level of SCG10 in the distal trunk of the injured sciatic nerves (*n* = 3 for each test, *P < 0.05).

GAP43 is a widely used marker of axonal regeneration after nerve injury (Ma et al., 2011). SCG10, which is expressed in adult sensory neurons, is an efficient and selective marker for regenerating sensory axons in the early stage of axonal regeneration (Shin et al., 2014). Both are barely detectable in intact adult nerves but are highly expressed in regenerating axons (Figures 1A–C,F–H). Figures 1B,C,F,G show that the GAP43- or SCG10-positive axons extending from the crush site to the distal trunk were significantly longer in the AA group than those in the saline group. This finding was verified by the quantification of Western blot analysis (Figures 1D,E or 1I,J). Significant differences among the groups were revealed by statistical analysis.

### AA Increases the Number of Regenerated Axons and the Level of Remyelination in the Injured Nerve

**Figure 2:**
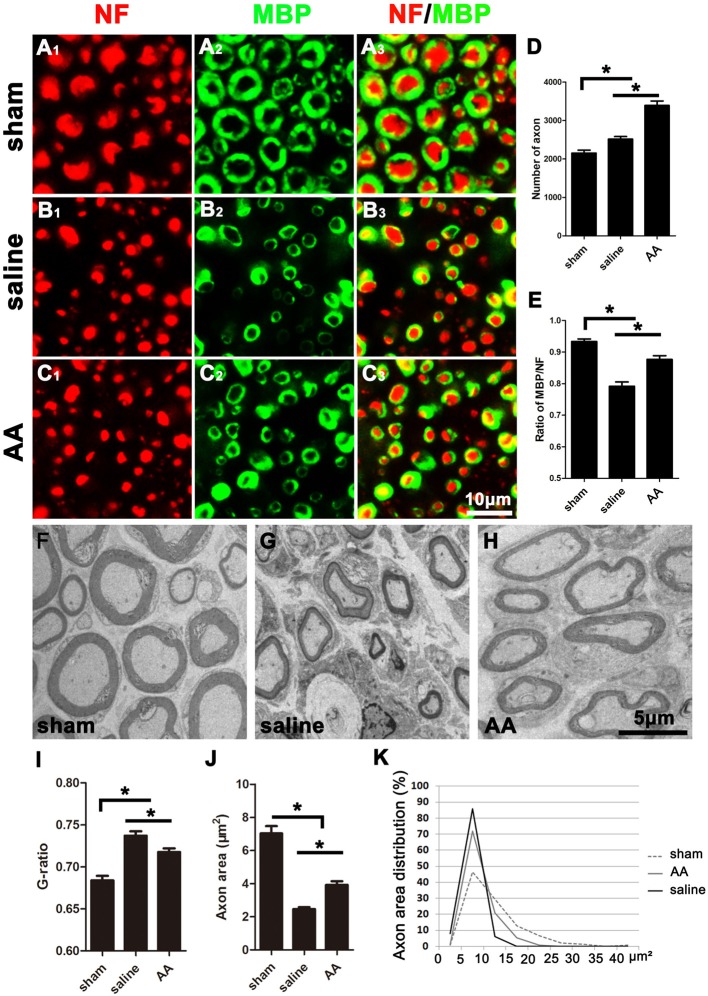
NF (Neurofilament, red) and MBP (Myelin Basic Protein, green) double immunohistochemistry and transmission electron microscopy (TEM) illustrating that AA enhances axonal regeneration and remyelination in the distal portion of the injured sciatic nerve at 28 dpi. Representative samples from **(A_1_–A_3_)** the sham group, **(B_1_–B_3_)** saline group, and **(C_1_–C_3_)** AA group. **(D)** The number of NF-positive axons in the cross-sections of the nerves (*n* = 6, *P < 0.05). **(E)** The MBP/NF ratio of myelinated axons from each group (*n* = 6, *P < 0.05). **(F–H)** Representative TEM images showing the axons and their myelin sheaths. **(I–K)** Quantification of the G-ratio **(I)**, the axonal area **(J)**, and the axonal area distribution (**K**; *n* = 4, *P < 0.05).

As shown in Figure 2, many NF-positive axons were present in the distal portion of the injured nerves at 28 dpi, and most of these axons were wrapped with MBP-positive myelin. Compared those in the saline group (Figure 2B), the number of axons (Figure 2D) and the ratio of MBP/NF (Figure 2E) were dramatically increased in the nerves of the AA group (Figure 2C). However, the number of axons and the thickness of the myelin sheaths in the nerves of the AA group did not reach the levels observed in the nerves of the sham control group (Figure 2A).

To accurately quantify the size of the regenerated axons and the myelin, parts of the nerves (*n* = 4 per group) were examined by TEM (Figures 2F–H). Statistically, the G-ratio (the diameter of the axon/the diameter of the whole nerve fiber), a widely used myelin thickness index (Tateshita et al., 2018), in the AA group was significantly lower than that in the saline group (Figure 2I), indicating that AA administration enhanced myelination and increased the thickness of the myelin sheaths. However, the G-ratio in the nerves of the AA group did not reach the level observed in the nerves of the sham group. In addition, the axonal area observed in the AA group was greater than that observed in the saline group (Figure 2J). The axonal area distribution (Figure 2K) reveals that the predominance of axons in the saline group had an area in the range of 0–15 μm^2^. Almost no axons in this group had an area greater than 15 μm^2^. Most axons in the AA groups had an area in the range of 0–15 μm^2^, but the percentage of axons with an area between 10 and 15 μm^2^ was notably higher in the AA group compared with that in the saline group (20.1% vs. 6.7%). Moreover, some axon areas (5.2%) in the AA group reached the range of 15–20 μm^2^. Overall, these data indicate that AA treatment not only increased the number and size of the regenerated axons but also enhanced the thickness of the myelin.

### AA Attenuates Myoatrophy of the Gastrocnemius Muscle

**Figure 3:**
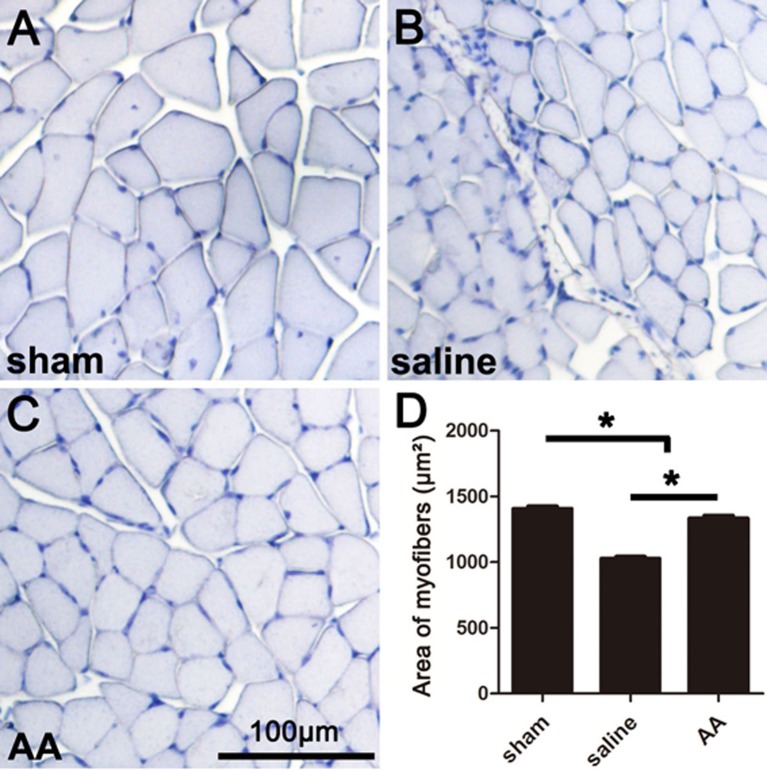
Histomorphometry of gastrocnemius muscles showing that AA alleviates myoatrophy in the target muscle. **(A–C)** Representative images of hemeatoxylin-stained transverse sections of gastrocnemius muscles from each group collected at 28 dpi. **(D)** Quantification of the myofiber area for each group (*n* = 10, *P < 0.05).

The gastrocnemius muscle is one of the main target muscles of the sciatic nerve and is widely used to reflect functional recovery in injured sciatic nerves. Compared to those in the sham group, the myofibers in the groups that underwent sciatic nerve injury (the saline group and the AA group) were notably smaller, which suggests dramatic myoatrophy. Nevertheless, the level of myoatrophy in the AA group was much less than that in the saline group (Figures 3A–C). This difference can be quantified by measurements and statistical analyses (Figure 3D).

### AA Enhances Motor and Sensory Function Recovery

**Figure 4:**
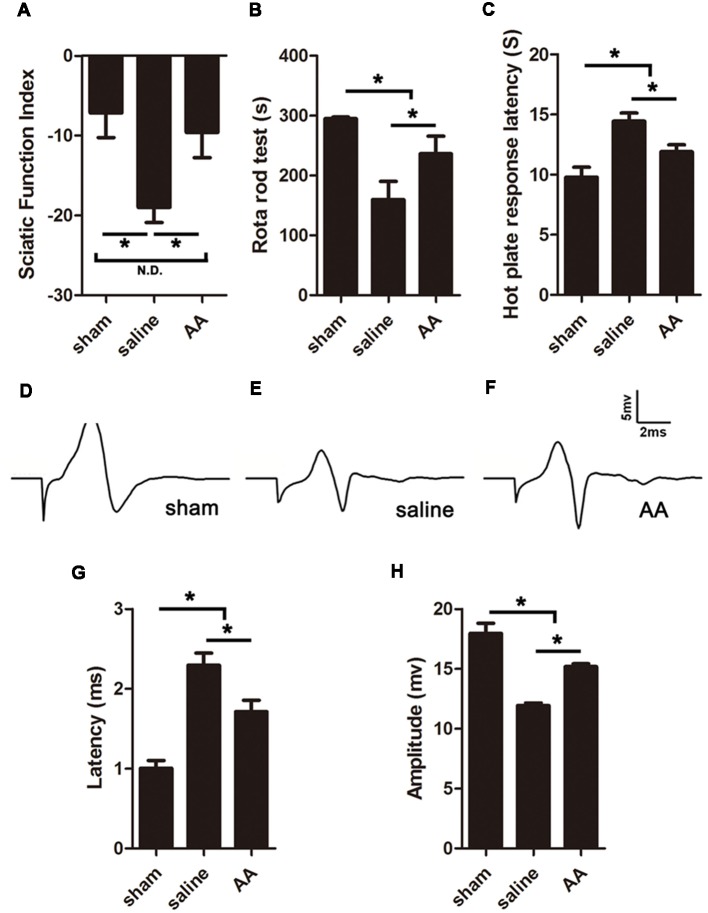
AA enhances functional recovery after sciatic nerve injury at 28 dpi. **(A)** Gait analysis results indicating the sciatic functional index (SFI). **(B)** Rotarod assay results showing the amount of time the mice remained on the rotating rod. **(C)** Hot plate test results showing withdrawal latency from a hot plate. **(D–F)** Respective compound muscle action potential (CMAP) images of the three groups. **(G,H)** Quantification of latencies and amplitudes of the CMAPs (*n* = 10, *P < 0.05; N.D. indicates no significant difference).

The SFI is among the most widely used parameters for measuring reflexive motor function after sciatic nerve injury. Compared to that in the sham group, the SFI value was significantly decreased in the PNI groups. However, this lower SFI was dramatically reversed by AA treatment (Figure 4A). Motor coordination and balance were also measured by a rotarod test. The mice in the AA group remained on the rotating rod for a much longer duration than the mice in the saline group but for a shorter duration than the mice in the sham group (Figure 4B).

Furthermore, sensory functional recovery was assessed with the hot plate test, in which longer latency reflexes suggest a sluggish sensory function in the tested foot. As expected, sciatic nerve injury caused the latency to significantly increase in the saline group compared to that in the sham group, but AA treatment shortened the latency (Figure 4C).

### AA Facilitates Nerve Conduction Function Recovery

The amplitude and latency of the CMAP were measured by electrophysiology. A shorter latency corresponds to quicker nerve conduction, which may result from a higher level of nerve myelination. A higher amplitude indicates a greater number of regenerated axons and a higher level of reinnervation of the measured muscles (Park et al., 2018). Quantification analysis of the CMAP images (Figures 4D–F) revealed that the latency in the sham group < AA group < saline group (Figure 4G) and that the amplitude in the sham group > AA group > saline group (Figure 4H). The differences among the groups were all statistically significant. Overall, the data from the above experiments indicate that PNI impairs nerve conduction, while the oral administration of AA significantly accelerates functional recovery, including the recovery of motor and sensory behavior and nerve conduction electrophysiology.

### AA Promotes Neurite Outgrowth and Alleviates RhoA Expression in Cultured DRG Neurons

**Figure 5:**
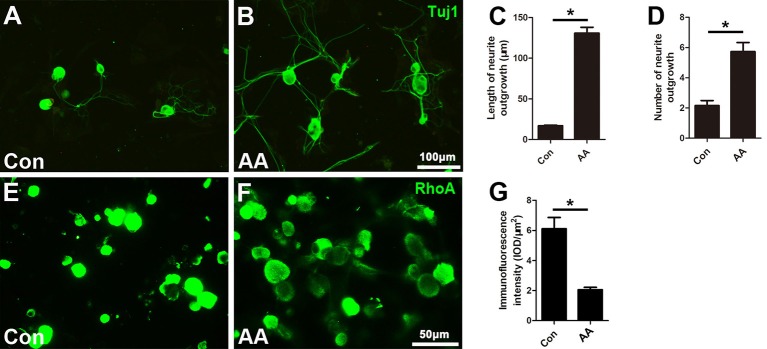
AA promotes neurite outgrowth and alleviates RhoA expression in cultured dorsal root ganglia (DRG) neurons. **(A,B)** Representative images of cultured neurons immunostained for Tuj1 to show the neurites. **(C,D)** Comparison of neurite length and number between two groups is shown (*n* = 3, *P < 0.05). **(E–G)** Immunofluorescence and quantification analysis illustrated that the immunointensity of RhoA was significantly decreased in the AA group (*n* = 3, *P < 0.05).

When the isolated DRG neurons were cultured in medium with a low concentration of serum (1% FBS), there were few neurites extending from the soma, and those that were present were short in length, as illustrated by Tuj1 immunostaining (Figure 5A). AA treatment dramatically increased the number and length of the neurites (Figures 5B–D). Moreover, immunostaining showed that RhoA immunoreactivity was concentrated in the neuronal soma and that the immune intensity was much higher in the control group than in the AA group (Figures 5E–G).

### AA Promotes Proliferation, Phagocytosis and Neurotrophic Factor Secretion in SCs

**Figure 6:**
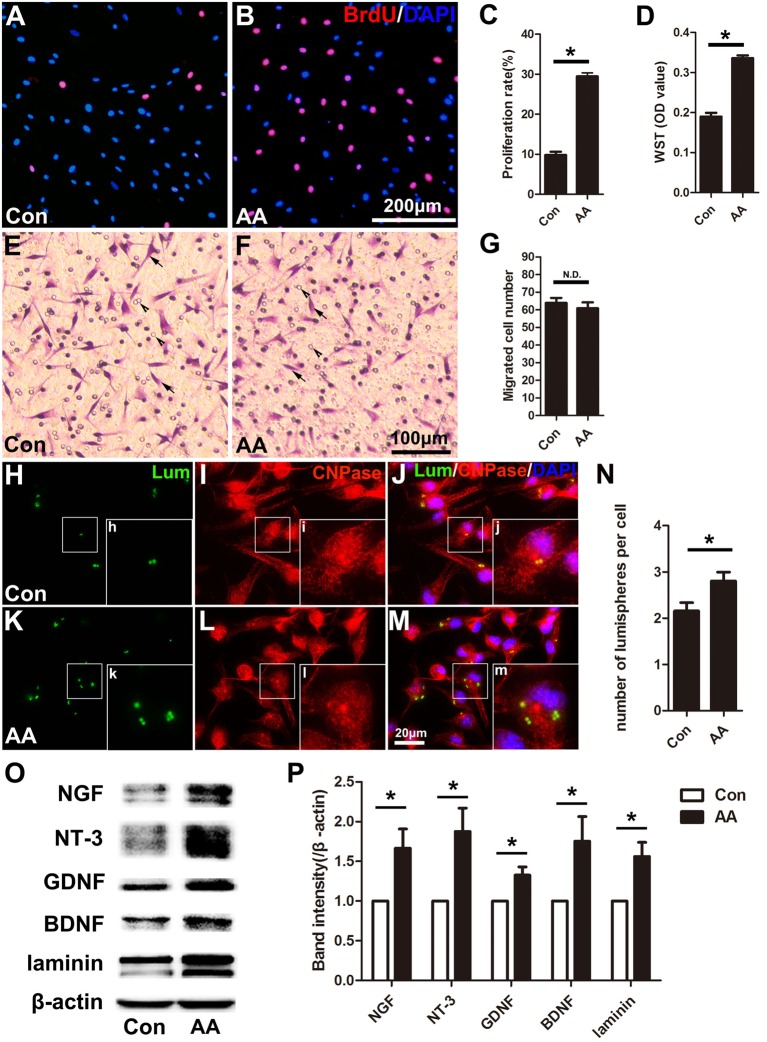
AA promotes proliferation, phagocytosis and neurotrophic factor secretion in Schwann cells (SCs) but does not affect the migration of SCs *in vitro*. **(A,B)** Representative images of BrdU immunofluorescence staining from each group. The total cells were labeled with DAPI (blue). **(C,D)** Quantitative analysis of the BrdU and WST assays (*n* = 3, *P < 0.05). **(E,F)** Representative images showing migrated SCs (arrows) from each group (arrowheads indicate the micropores on the membrane). **(G)** Statistics showing there was no significant difference between the two groups (*n* = 3, N.D. indicates no significant difference). **(H–M)** Images showing the ingested lumispheres (green) within the cells. The SCs were identified with CNPase immunocytochemistry (red). **(N)** Quantitative analysis of the number of lumispheres per cell (*n* = 3, *P < 0.05). **(O)** Western blots and **(P)** their quantification indicate the expression level of neurotrophic factors (*n* = 3, *P < 0.05).

**Figure 7:**
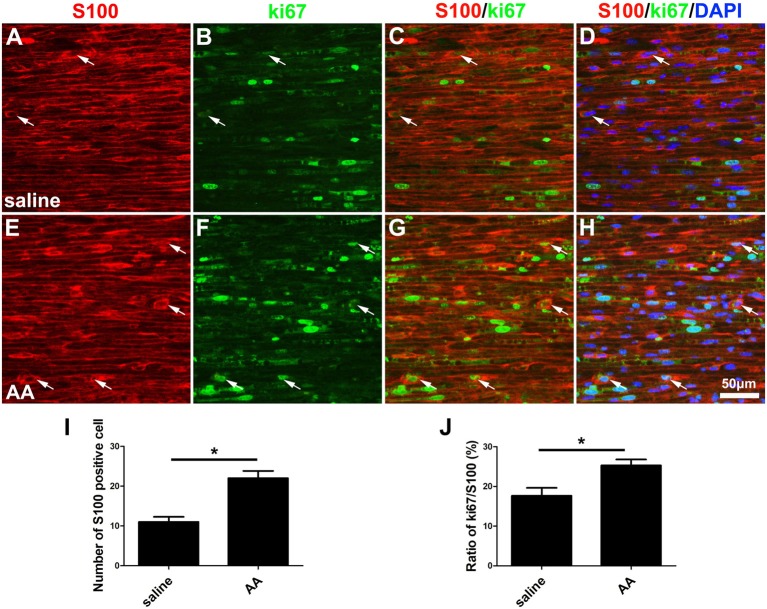
AA promotes the proliferation of SCs *in vivo*. **(A–H)** Representative images of S100 (red) and ki67 (green) double immunostaining showing proliferating SCs (arrows) in each group. **(I)** Quantitative analysis of the number of S100-positive cells (*n* = 3, *P < 0.05). **(J)** The ratio of ki67/S100 in each group (*n* = 3, *P < 0.05).

In the present study, two methods were used to assess the capability for cell proliferation *in vitro*. Using a BrdU assay, we found that the ratio of BrdU-positive cells in the AA-treated SCs was significantly higher than that in SCs from the control group (Figures 6A–C). The OD value determined by the WST-1 test in the AA group was also statistically higher than that in the control group (Figure 6D). The above data indicate that AA can increase the proliferation of SCs. To confirm the results obtained from the *in vitro* experiments, S100 and Ki67 double immunohistochemistry was performed on the longitudinal sections of the sciatic nerves collected at 3 dpi (Figures 7A–H). Quantitative analysis illustrated that both the number of S100-positive cells (Figure 7I) and the ratio of ki67/S100 (Figure 7J) in the AA group were higher than those in the saline group. However, the Transwell test indicated that the migration ability of the SCs was not affected by AA, as the number of migrated cells was not different between the AA group and the control group (Figures 6E–G). Since the lumispheres are considered foreign materials to the co-cultured cells, it is expected that some of them would be ingested by SCs (Figures 6H–M); thus, the number of lumispheres in each cell reflects the cell’s phagocytic capability. Quantitative analysis illustrated that there were more ingested lumispheres in the AA-treated SCs than in SCs from the control group (Figure 6N). Finally, Western blotting indicated that the protein levels of NGF, NT-3, GDNF, BDNF, and laminin expressed by the SCs were all significantly increased by AA treatment (Figures 6O,P).

### AA Increases the Proliferation, Migration, Phagocytic Capability and M2 Subtype Polarization of Macrophages

**Figure 8:**
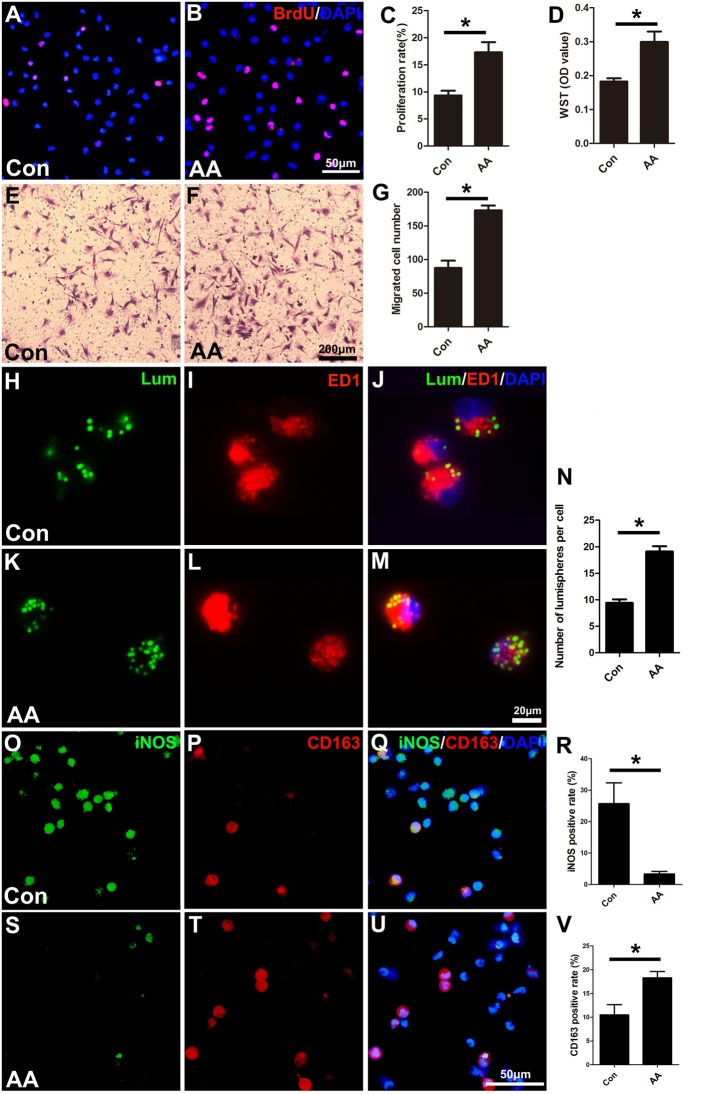
AA promotes the proliferation, migration, phagocytic capability, and M2 polarization of macrophages but inhibits M1 polarization *in vitro*. **(A,B)** Images of immunofluorescence staining for BrdU (red). Cell nuclei were labeled with DAPI (blue). **(C,D)** Statistical analysis of the quantifications of the BrdU and WST assays (*n* = 3, *P < 0.05). **(E–G)** Images and quantification showing migrated macrophages in the two groups (*n* = 3, *P < 0.05). **(H–M)** Representative images of lumispheres ingested by macrophages (ED1 positive). **(N)** Quantitative analysis of the number of lumispheres per macrophage (*n* = 3, *P < 0.05). **(O–U)** Representative images and quantitative analysis illustrate that, compared with that in the control group, the number of M1-type macrophages in the AA group was reduced **(R)**, while the number of M2 macrophages in the AA-treated group was increased (**V**; *n* = 4, *P < 0.05).

**Figure 9:**
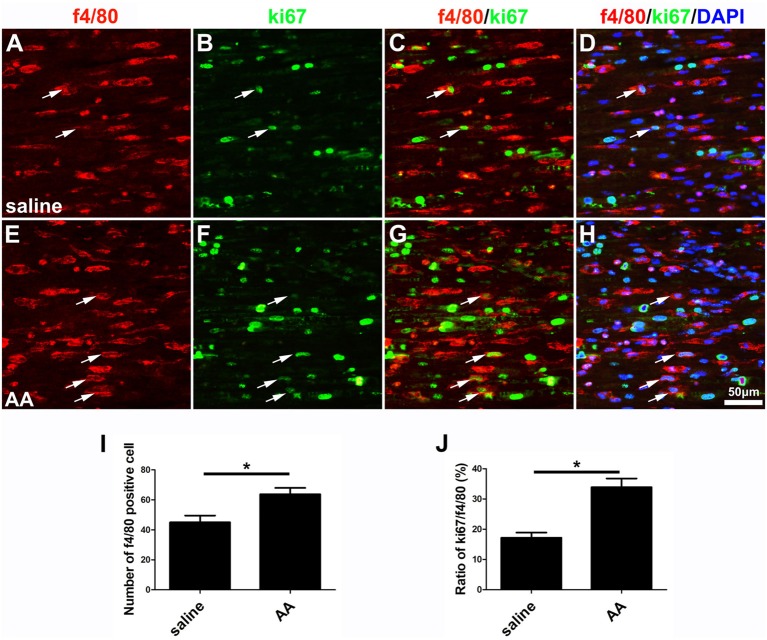
AA promotes the proliferation of macrophages *in vivo*. **(A–H)** Representative images of f4/80 (red) and ki67 (green) double immunohistochemistry showing proliferating macrophages (arrow) in each group. **(I)** Quantitative analysis of the number of f4/80-positive cells (*n* = 3, *P < 0.05). **(J)** The ratio of ki67/f4/80 in each group (*n* = 3, *P < 0.05).

As described in the “Materials and Methods” section, the proliferation, migration and phagocytic capability of the cultured macrophages were detected using the same assays as those used in the SCs. Statistical data of the BrdU assay (Figures 8A–C) and the WST-1 test (Figure 8D) showed that the BrdU positive ratio and the WST-1 OD value were significantly higher in the AA group compared to those in the control group, which indicated that AA increases the proliferation of macrophages. Double immunohistochemistry with f4/80 and ki67 antibodies of the sciatic nerve longitudinal sections (Figures 9A–H) illustrated that both the number of f4/80-positive cells (Figure 9I) and the ratio of ki67/f4/80 (Figure 9J) in the AA group were higher than those in the saline group. Subsequently, the migration and phagocytic capability of the macrophages were detected by a Transwell assay and lumisphere ingestion test in the cultured cells. The number of macrophages on the lower surface of the Transwell membrane was almost double that in the AA group (Figures 8E–G). This upregulation effect was also found in the phagocytic capability assay (Figures 8H–M). The number of lumispheres ingested by the macrophages was almost doubled by treatment with AA (Figure 8N).

**Figure 10:**
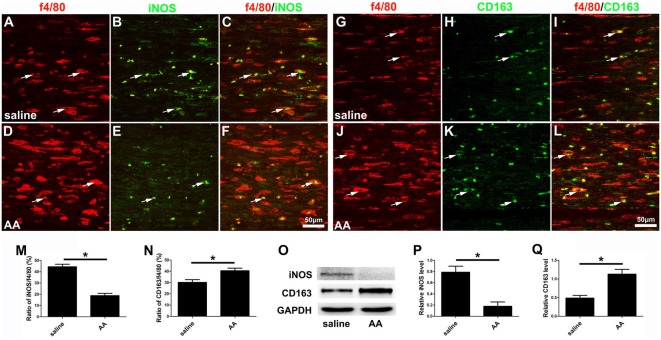
AA inhibits the M1 polarization of macrophages but promotes M2 polarization *in vivo*. **(A–F)** Representative images of f4/80 (red) and iNOS (green) double immunohistochemistry in each group. **(G–L)** Representative images of f4/80 (red) and CD163 (green) double immunohistochemistry in each group. **(M)** The ratio of iNOS/f4/80 in each group (*n* = 3, *P < 0.05). **(N)** The ratio of CD163/f4/80 in each group (*n* = 3, *P < 0.05). **(O–Q)** Western blots and schematic diagrams of the statistical analysis showing the protein levels of iNOS and CD163 in the distal trunk of the injured sciatic nerves (*n* = 3, *P < 0.05).

Finally, macrophage polarization was assessed by immunocytochemistry with specific markers of a pro-inflammatory subtype (M1, iNOS) or an anti-inflammatory subtype (M2, CD163), both *in vitro* (Figures 8O–V) and *in vivo* (Figures 10A–L). Quantitative results illustrated that the number of iNOS-positive M1 macrophages (Figures 8R, 10M) decreased dramatically, while the number of CD163-positive M2 macrophages (Figures 8V, 10N) increased significantly in the AA group. The Western blot results (Figures 10O–Q) were consistent with the immunostaining data.

## Discussion

PNI is a common disease worldwide. Epidemiologic studies have indicated that PNI is involved in up to 2.8% of trauma cases (Noble et al., 1998). PNI has a marked impact on patient quality of life because the injury causes partial or complete loss of function in the target organs of the lesioned nerve. In contrast to the central nervous system (CNS), where damaged axons are usually unable to regenerate, axons in the PNS are capable of regeneration in cases where the injured nerve is properly surgically repaired (Gey et al., 2016; Wang et al., 2017). Nevertheless, the regeneration rate of peripheral nerves is slow, and functional recovery is generally far from satisfactory (Modrak et al., 2017). Thus, the effort to identify effective and safe drugs that can accelerate the neural regeneration rate has gained broad awareness.

To the best of our knowledge, this report is the first to provide solid evidence demonstrating that the oral administration of AA has significant efficacy in facilitating peripheral nerve regeneration. Previously, some studies have reported using vitamins to treat peripheral nerve injuries, but these studies mainly focused on nerve injury-derived neuropathic pain, and the reported effects were mainly the results of interactions between the vitamins and pain-related receptors in the spinal cord (Teixeira et al., 2002; Lu et al., 2011). In addition, many studies have demonstrated that AA plays a role in myelin formation, mainly myelination studies of SC/neuron coculture systems *in vitro* (Podratz et al., 2001; La Marca et al., 2011; Hyung et al., 2015), and observations of peripheral nerve demyelination in Charcot-Marie-Tooth disease (Passage et al., 2004; Gess et al., 2013). However, no data revealing the role of AA in axonal regeneration and remyelination after PNI are available.

The present data showed that at 3 dpi, GAP43- or SCG10-positive regenerating axons in lesioned nerves treated with AA were significantly longer than those in nerves from the control group, which indicates that AA can accelerate early axonal regeneration. In addition, axons in the distal stump and their myelin sheaths must be disintegrated after nerve injury. Subsequently, the axons regrow from the proximal end, extend to the distal trunk and become remyelinated by SCs. Thus, the axons and myelin sheaths detected in the distal portion of an injured nerve are the result of nerve regeneration. As shown in Figure 2, the number of NF-positive axons in the AA group was greater than that in the saline group and even greater than that in the sham group. Following nerve injury, a single proximal axon generates several lateral buds and then regenerates toward the distal trunk during nerve repair. These lateral buds noticeably outnumber the original axons in the nerve. This phenomenon is known as “multiple amplification” (Jiang et al., 2007; Deng et al., 2017). The present assessments by NF/MBP double immunostaining and TEM demonstrate that AA may increase the number and size of the regenerated axons and the myelin thickness.

It is well known that PNI-derived denervation leads to myoatrophy in the target muscles. Once regenerating axons reach and reinnervate the target muscles, myoatrophy can be reversed (Pak et al., 2016). Here, we performed histomorphometry on the gastrocnemius muscle and found larger myofibers in the AA-treated animals compared with those in the saline-treated control animals. This result combined with the behavior, electrophysiology and morphology results, leads us to believe that supplementation with AA is an efficient approach for facilitating nerve regeneration in PNI.

The most disastrous consequences of PNI are the loss of motor and sensory functions in the area innervated by the injured nerve. Here, we utilized three widely used behavior tests to evaluate functional nerve regeneration. The data shown in Figure 4 demonstrate that all nerve injury-derived functional defects identified by comparing the saline group with the sham group were significantly alleviated in the AA group, which indicated that supplementation with AA can significantly improve both motor and sensory functional recovery. Then, the behavior profiles were confirmed by an electrophysiological assessment that showed that the AA treatment shortened the latency and increased the amplitude of CMAP in the injured sciatic nerves. CMAP is commonly used to measure nerve conduction properties. A shortened latency can reflect quicker nerve conduction, which is mainly related to the level and quality of myelin surrounding the nerve fibers. In addition, an increased amplitude suggests that a greater number of regenerated axons are present at the assessed time point (Wang et al., 2015; Salehi et al., 2018). Overall, the data derived from this electrophysiological assessment illustrate that nerve conduction capability is improved by treatment with AA.

Subsequently, three types of cells, including neurons, SCs and macrophages, which are involved in nerve injury and repair, were investigated to explore the potential role of AA in nerve regeneration. First, we isolated neurons from the DRG after axon removal, and the neurons were cultured in medium with a low concentration of FBS (1%) to mimic an unhealthy microenvironment. In these cultures, we found that AA significantly increased the number and length of regenerated neurites and decreased the expression of RhoA. RhoA is a crucial inhibiting factor for neuronal regeneration and is highly expressed in injured neurons (Devaux et al., 2017; Hu et al., 2017). Therefore, the downregulation of RhoA expression is beneficial to neurite outgrowth.

In addition to neuronal intrinsic capacity, environmental factors such as SC activation can contribute to axonal regeneration and remyelination. Adequate proliferation to produce a sufficient cell population and the migration of cells to the correct site are important processes in peripheral nerve regeneration even before the cells contribute to regeneration (Zhang et al., 2017). The present *in vitro* data from the BrdU assay and the WST-1 assay and the *in vivo* data from the double immunohistochemistry for S100 and ki67 demonstrated that AA can promote SC proliferation but has no statistical effect on SC migration. SCs are reported to participate in myelin phagocytosis in the early stage of Wallerian degeneration, prior to the recruitment of macrophages (Lutz et al., 2017). Using a lumisphere test, we found that many more lumispheres were ingested by AA-treated SCs than by naïve cells. These results indicate that AA can enhance the phagocytic capability of SCs and favor nerve regeneration by accelerating the elimination of debris from the injured nerve. Another important function of SCs is the secretion of neurotrophins and extracellular molecules (ECMs) to support and induce axonal regeneration. Herein, we detected the expression levels of laminin, a well-accepted component of the ECM that is essential for neuroregeneration, and NGF, NT-3, GDNF and BDNF, which are neurotrophins produced by SCs. Consistently, these proteins were all significantly upregulated by AA treatment. Overall, our data suggest that AA may affect the behavior of SCs and thereby enhance nerve regeneration.

Macrophage recruitment to the injured nerve is considered a key prerequisite for nerve regeneration by eliminating broken myelin and axonal debris. Our *in vitro* data also demonstrate that AA promotes the phagocytic and migration capabilities of macrophages. Furthermore, the present *in vitro* and *in vivo* data indicate that AA may significantly improve the proliferation and M2 polarization while decreasing the pro-inflammatory subtype (M1) polarization of macrophages. The anti-inflammatory subtype (M2) of macrophages is well accepted and has the capability to facilitate axonal regeneration by secreting axonal regeneration-related factors, including ECM proteins, growth factors, cytokines and chemokines (Gaudet et al., 2011; Nadeau et al., 2011). In this view, our data are reasonable. However, post-injury inflammatory signals are also regarded as having a positive influence on axonal oxidative signaling for promoting axonal regeneration (Hervera et al., 2018). As inflammatory signals are mainly released by M1 macrophages, the present data cannot thoroughly explain the potential effect of the decreased number of M1 macrophages induced by treatment with AA in nerve regeneration. We hypothesize that an appropriate ratio of M1 vs. M2 macrophages may be important for nerve regeneration, and this hypothesis should be systematically studied in the future. In summary, the present experiments suggest that AA may have an effect on neurons, SCs and macrophages to facilitate nerve regeneration.

## Conclusion

The current study is the first to provide experimental evidence demonstrating that supplementation with AA can sufficiently promote nerve regeneration in a model of moderate nerve injury and that this effect is related to the effect of AA on neurons, SCs and macrophages, which are key cells involved in nerve injury and repair. Given that AA is an essential nutrient in our diet and that it has been widely used as a dietary supplement and clinical drug (Chin and Ima-Nirwana, 2018; Granger and Eck, 2018), supplementation with AA may be a safe, affordable, convenient, and efficient strategy for accelerating peripheral nerve regeneration.

## Author Contributions

JG designed the study. LL, YL, ZF and XW performed the experiments. LL, YL, ZF, ZL, JW, JD, DT, MP and XH participated in collecting and analyzing experimental data. HZ, XH, and ML contributed to the double-blind analyzing data and statistics. The manuscript was written by JG, LL and reviewed by all authors.

## Conflict of Interest Statement

The authors declare that the research was conducted in the absence of any commercial or financial relationships that could be construed as a potential conflict of interest.
